# Open Midline Decompression with Ligament Reconstruction for Multiple-Level Spinal Stenosis in Elderly Patients

**DOI:** 10.3390/neurosci6010018

**Published:** 2025-02-25

**Authors:** Shin-Jae Kim, Sang-Ho Lee, Junseok Bae

**Affiliations:** Department of Neurosurgery, Chungdam Wooridul Spine Hospital, Seoul 06068, Republic of Korea; nsdoc@wooridul.co.kr (S.-J.K.); sh500909@wooridul.co.kr (S.-H.L.)

**Keywords:** open midline decompression, ligament reconstruction, multiple spinal stenosis, soft stabilization

## Abstract

(1) Background: Multiple lumbar spinal stenosis (LSS) is a degenerative disease that is increasingly prevalent with global aging. Multilevel fusion surgery is burdensome to perform in elderly patients, especially with osteoporosis and underlying disease. This study introduces open midline decompression (OMD) with ligament reconstruction as an alternative stabilization technique for elderly patients with multilevel LSS. (2) Methods: A retrospective review included 42 elderly patients aged 70 or older diagnosed with LSS at three or more levels and who underwent OMD with ligament reconstruction. Pre- and postoperative clinical and radiologic data were analyzed. (3) Results: Thirty-three patients underwent three-level surgeries, and twelve patients underwent four-level surgeries. The mean operative time was 240 ± 42.2 min (74.6 ± 14.9 min per level) with a mean blood loss of 282.9 ± 167.1 cc. Clinical outcome (VAS) and quality of life parameters (SF-12) showed significant improvement after surgery. Postoperative MRI showed sufficient decompression. Dynamic X-rays showed improvement in instability after surgery, but it was statistically insignificant. (4) Conclusions: OMD with ligament reconstruction provides effective neural decompression while preserving the posterior arch and offers soft stabilization with artificial ligaments. It is a safe and viable surgical option for elderly patients with multilevel LSS.

## 1. Introduction

The modern world has transformed into an aging society, where the time individuals spend in old age has substantially increased. Consequently, the number of people affected by lumbar spinal stenosis (LSS), a degenerative condition that typically becomes more prevalent after the age of 50, is rising dramatically. Studies report that the prevalence of LSS in the general population can reach as high as 11% [1]. Furthermore, LSS is recognized as the leading cause of spinal surgery in patients over 65 years of age [2].

Patients diagnosed with multiple levels of LSS often visit medical facilities with symptoms such as chronic back pain, leg numbness, and neurogenic claudication. They are generally managed with a range of conservative treatments, including structured exercise programs, pharmacological interventions, physical therapy, steroid injections, or epidural neurolysis. Historically, many elderly patients managed their pain using these conservative methods for the remainder of their lives [3]. However, with the increasing life expectancy of older adults [4], the importance of improving quality of life (QOL) has become more significant than ever. In this context, surgery has emerged as a more proactive approach to achieving better outcomes for elderly individuals [5]. Nevertheless, elderly patients frequently have significant comorbidities [6,7], making prolonged surgical procedures under general anesthesia particularly burdensome. Multilevel LSS, in particular, often presents with spinal instability that requires fixation and stabilization. For this reason, treatment for multiple levels of LSS in older adults has traditionally been either not recommended or limited to decompression alone, without addressing stabilization.

The development of minimally invasive surgical (MIS) techniques, such as tubular or endoscopic decompression [8,9,10,11], has been a game-changer, providing less invasive options for addressing LSS. However, these approaches are not without limitations, particularly when dealing with elderly patients suffering from multilevel LSS accompanied by instability. Some authors have proposed alternative decompression techniques, including spinous splitting laminectomy [12,13] and contralateral decompression methods [14,15]. While these techniques can be beneficial, decompression alone may lead to long-term instability, contributing to recurrent stenosis or persistent back pain [16].

To address these challenges, soft stabilization techniques using artificial ligaments have been developed. These aim to support the spine while preserving segmental motion. Artificial ligaments are already widely used in orthopedic surgeries, particularly for knee and shoulder joints. However, in the field of spine surgery, fusion procedures remain predominant, and soft stabilization treatments utilizing artificial ligaments have only recently started to gain attention. Several studies have demonstrated promising clinical and radiological outcomes following ligamentoplasty for stabilizing decompressed spinal segments [17,18,19,20]. The artificial ligament used in this study, SHLee Ligament™ (Prestigemedicare, GEMSKOREA, Seoul, Republic of Korea), is primarily composed of polyethylene terephthalate and has been approved by the MFDS (the Ministry of Food and Drug Safety in Korea).

In this study, the authors employed microscopic open midline decompression (OMD), a technique that achieves lumbar spinal canal decompression by removing the thickened ligamentum flavum (LF) and decompressing the lateral recess through an interspinous window. This approach minimizes invasiveness by preserving the facet joint complex and spinous–laminar arch, leading to reduced intraoperative bone bleeding and a lower risk of iatrogenic instability. Additionally, combining OMD with ligamentoplasty has proven particularly advantageous for elderly patients with multilevel LSS and accompanying instability.

Lee et al. [21] previously demonstrated the clinical effectiveness of ligamentoplasty combined with posterior fusion surgery in 2005. Building on this foundation, the present study aims to detail the technical aspects of OMD with ligament reconstruction while reporting the outcomes of multilevel surgeries performed on elderly patients.

## 2. Materials and Methods

### 2.1. Study Design and Population

A comprehensive retrospective review was conducted on elderly patients aged 70 years or older who had been diagnosed with lumbar spinal stenosis (LSS) involving three or more spinal levels. These diagnoses included cases with or without spinal instability. The patients underwent open midline decompression (OMD) combined with ligament reconstruction between January 2019 and December 2019. The surgical procedures were performed on individuals who continued to report persistent symptoms, such as chronic pain and functional impairments, even after an extended period of sufficiently conservative treatments.

Patients with severe degenerative spondylolisthesis of grade 2 or higher [22] or significant spinal instability—defined as a sagittal angulation angle exceeding 15° at the L1–2, L2–3, and L3–4 levels; 20° at the L4–5 level; and 25° at the L5–S1 level [23,24]—were excluded from this study to ensure optimal surgical outcomes. Comprehensive preoperative evaluations included assessments of sex; age; smoking history; and pre-existing medical conditions such as hypertension, diabetes, heart disease, liver disease, brain disease, lung disease, and cancer, among others (Table 1).

Additional clinical data were collected to assess bone mineral density (BMD), preoperative visual analog scale (VAS) scores for pain, and the 12-item short-form survey (SF-12) scores [25] (Table 2). Operational metrics such as the levels operated on, surgical duration, intraoperative blood loss, and postoperative complications were meticulously documented. Further clinical data included the duration of hospital admission; postoperative VAS scores at 1 week, 1 month, and 6 months; as well as SF-12 scores at 6 months postoperatively.

To evaluate the effectiveness of the surgical decompression, pre- and postoperative magnetic resonance imaging (MRI) scans were compared on postoperative day 1 to confirm sufficient nerve root decompression. Additionally, dynamic lumbar X-rays captured during flexion were used to assess changes in spinal stability before and one week after the surgery. These evaluations aimed to provide a comprehensive understanding of both the immediate and intermediate-term outcomes of OMD combined with ligament reconstruction in this elderly population.

### 2.2. Ethics Statements

All patients included in this study participated voluntarily and provided written informed consent after receiving a comprehensive explanation of the procedure. This explanation included detailed information about both the potential risks and anticipated benefits of the treatment. This study received formal approval from the Chungdam Wooridul Spine Hospital institutional review board (2021-01-WSH-001) of the conducting institution, ensuring compliance with all necessary ethical requirements.

The research protocol adhered strictly to the principles outlined in the Declaration of Helsinki, which serve as a cornerstone for ethical medical research worldwide. Additionally, this study followed the Korea Good Clinical Practice (KGCP) guidelines, further guaranteeing that all processes met high standards of patient safety, ethical integrity, and clinical quality.

### 2.3. Surgical Technique

- Open Midline Decompression

**Figure 1:**
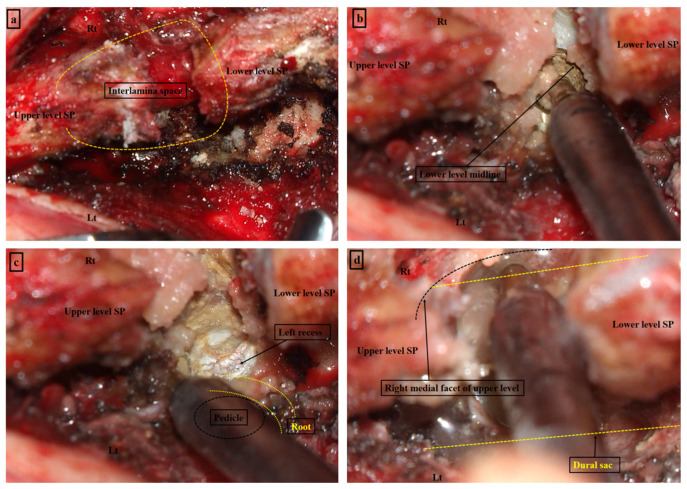
Process of open midline decompression. (**a**) Muscle resection is performed bilaterally from the upper level spinous process (SP) to the lower level SP. (Yellow line—Lateral margin of Interlamina space) (**b**) Lower level midline drilling is conducted to reveal the dural sac. (**c**) The lower level recess is drilled until the medial wall of the pedicle is detached. (Yellow line—Lateral margin of Root) (**d**) Upper level medial facet drilling is performed with minimal facet destruction by using the undercutting drilling technique from the contralateral side via the “interspinous window”. (Yellow line—Lateral margin of Dural sac) Source: own composition.

**Figure 2:**
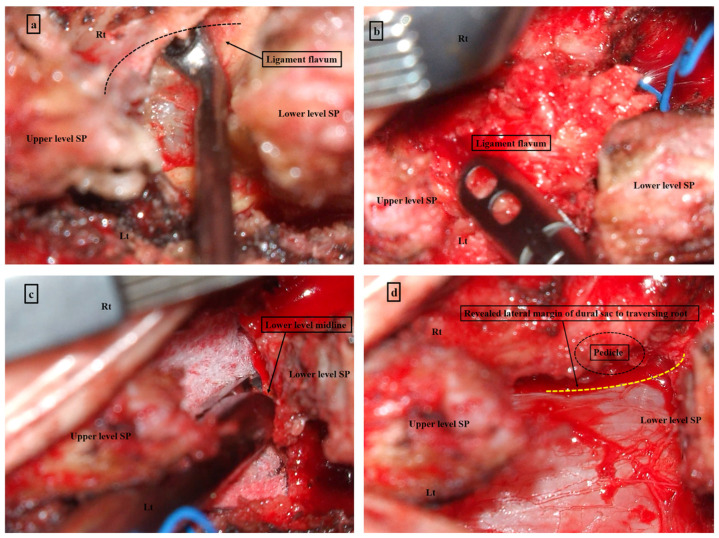
Process of open midline decompression continued. (**a**) After sufficient drilling, the ligamentum flavum is detached from the lamina by gently using a curette. (**b**) Flavectomy is performed with en bloc removal. (**c**) Additional decompression of the upper and lower laminae above the dural sac is conducted. (**d**) It is confirmed that the dural sac and both traversing roots are sufficiently decompressed. Source: own composition. (Yellow line—Revealed lateral margin of dural sac to traversing root).

Under general anesthesia, the patient was placed in the prone position using a Wilson frame. Sterile skin preparation and surgical draping were performed. A midline skin incision was made from the center of the upper spinous process to the lower margin of the lower spinous process at the index level (Figure 1a). The fascia was incised 0.5 cm from the midline on the left side. After para-spinal muscle dissection was conducted on the left side, the supraspinous and interspinous ligaments were detached from the spinous process using mono-polar cautery. The contralateral para-spinal muscle was dissected using a detached ligament complex. A self-retraining retractor was placed at the level of the interspinous space. The “interspinous window” was prepared by cleaning the soft tissue using mono-polar cautery and a high-speed drill (Figure 1a). Under the microscopic view, decompression was performed from the caudal spinous base using a high-speed drill (Figure 1b), expanding laterally to the medial pedicle (Figure 1c). Cranial decompression was conducted by undercutting the cranial lamina using a high-speed drill. Medial facetectomy was also performed to decompress the lateral recess area by contralateral decompression (Figure 1d). Further decompression of the neural foramen could be conducted in cases of foraminal stenosis. Contralateral decompression was effective in preserving the facet joint during decompression of the lateral recess and foramen. It is important to preserve the integrity of the spinolaminar junction to avoid spinous fracture after decompression. En bloc flavectomy was performed by detaching the LF from the cranial and caudal attachments to the lamina (Figure 2a,b). After removing the hypertrophied LF, additional bony decompression of the upper and lower laminae above the dural sac was conducted (Figure 2c) to ensure that the dural sac and both traversing roots were sufficiently decompressed (Figure 2d).

- Ligament Reconstruction

**Figure 3:**
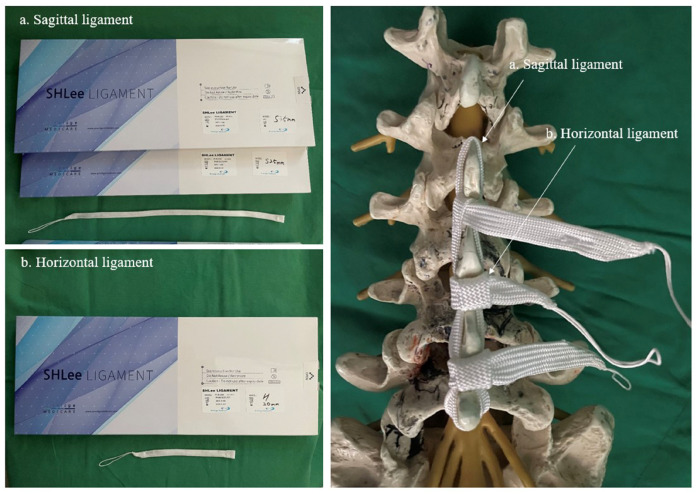
SHLee Ligament™ (Prestigemedicare, GEMSKOREA, Republic of Korea) consists of two main components. (**a**) Sagittal Ligament connects the spinous processes. (**b**) Horizontal Ligament bridges the interlaminar spaces. Source: own composition.

SHLee Ligament™ (Prestigemedicare, GEMSKOREA, Republic of Korea) consists of two main components: the “Sagittal Ligament”, which connects the spinous processes, and the “Horizontal Ligament”, which bridges the interlaminar spaces (Figure 3). Each end of the ligament features a detachable needle loop and a tunnel through which the ligament can pass. This design allows for a secure binding method by threading the ligament through these components during the procedure.

**Figure 4:**
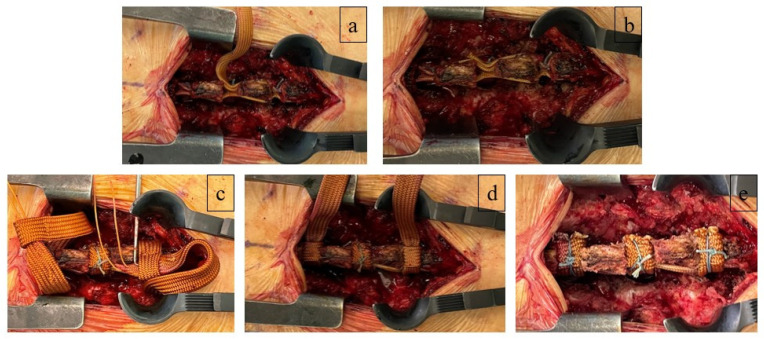
Process of SHLee ligament reconstruction. (**a**) The Sagittal Ligament was tied longitudinally between the spinous processes at the index level. (**b**) To prevent excessive spinal extension, 2-0 nylon sutures were placed near the center of the knot. (**c**) The Horizontal Ligament was then wound transversely around the interlaminar space, encasing the Sagittal Ligament. (**d**) The Horizontal Ligament was subsequently tightened. (**e**) Additional 2-0 nylon sutures were applied for reinforcement. Source: own composition.

**Figure 5:**
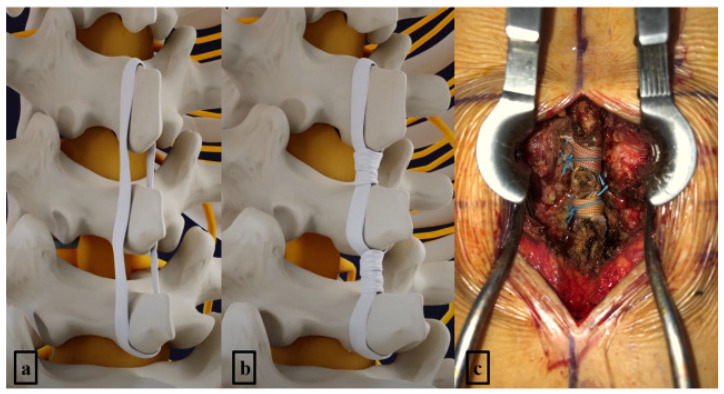
Process of SHLee ligament reconstruction. (**a**) The longitudinal component of the artificial ligament is tied at the index level of the spinous processes. (**b**) The horizontal component of the artificial ligament is tied around the longitudinal component at the interspinous spaces. (**c**) Surgical field of two-level ligament reconstruction after open midline decompression. Source: own composition.

After achieving sufficient hemostasis, the Sagittal Ligament was tied longitudinally between the spinous processes at the index level (Figure 4a). Prior to tightening the ligament with a contractor device, the regional lordotic angle of the lumbar spine was adjusted by altering the operating table’s angle. To prevent excessive spinal extension, 2-0 nylon sutures were placed near the center of the knot (Figure 4b). The Horizontal Ligament was then wound transversely around the interlaminar space, encasing the Sagittal Ligament (Figure 4c), and subsequently tightened (Figure 4d). Additional 2-0 nylon sutures were applied for reinforcement (Figure 4e). The surgical wound was closed upon completion of the procedure. Figure 5 illustrates a 3D representation of how the artificial ligament is secured post-surgery (Figure 5). This image provides a detailed view of the positioning and binding mechanism of the ligament, demonstrating its configuration between the spinous processes and within the interlaminar space.

## 3. Results

**Figure 6:**
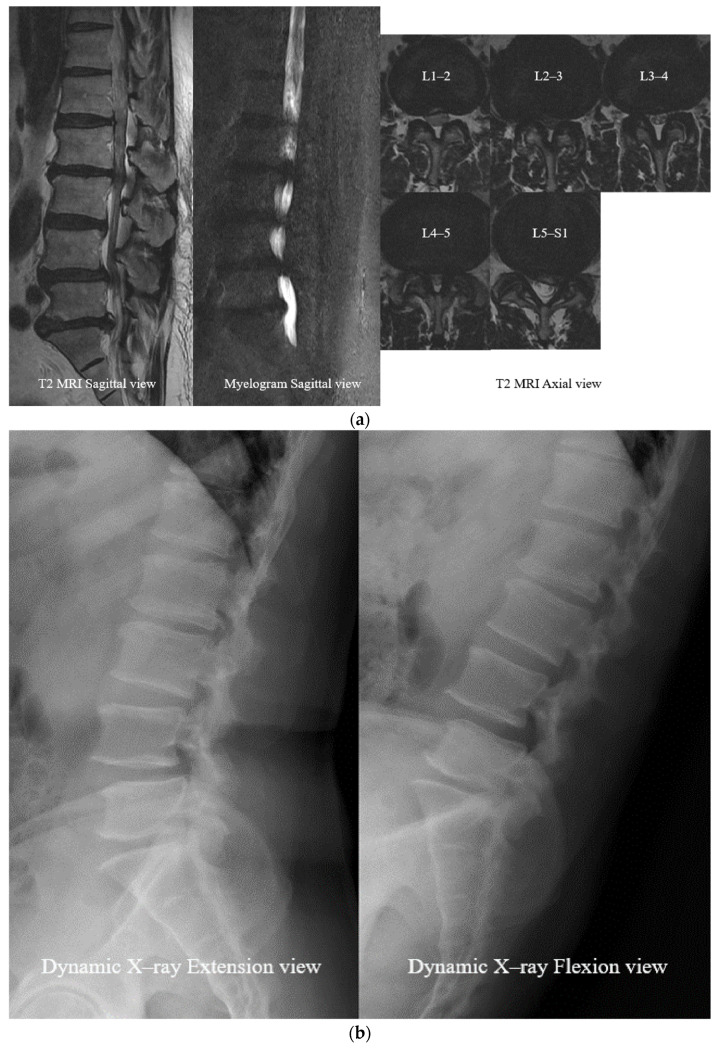

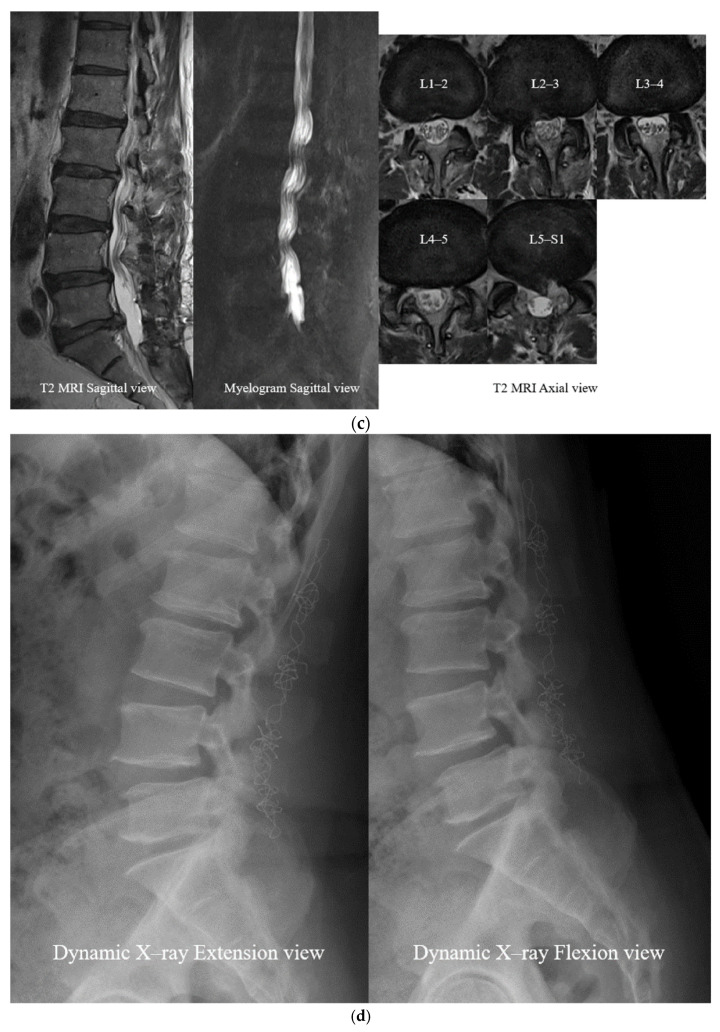

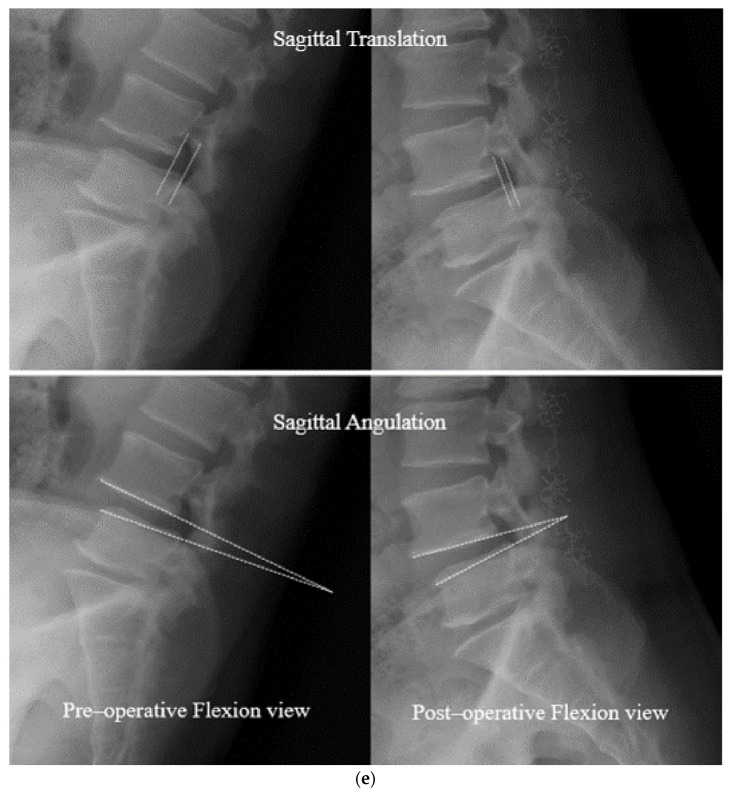
Case 1. An 82-year-old male patient suffered from low back pain, sacral pain on both sides, and numbness in both legs for 3 years. (**a**) Preoperative MRI and myelogram: spinal stenosis at L1 to L5. (**b**) Preoperative dynamic X-ray: instability at L4–5. (**c**) Postoperative MRI: well-decompressed dural sac. (**d**) Postoperative dynamic X-ray: instability improvement at L4–5. (**e**) Sagittal translation decreased from 9.1 mm to 5.2 mm at L4–5. Sagittal angulation increased from 5.8° to 11.4°. Source: own composition. The small white dot line represents an extension of the posterior wall of the vertebra body and indicates the degree of listhesis. The large white dot line is an extension of the endplate line of the vertebra body and represents the degree of angulation.

Overall, 18 men and 27 women underwent OMD with ligament reconstruction between January 2019 and December 2019. Patients’ mean age was 76.3 (±3.79) years and mean BMI was 24.97 (±3.16) kg/m^2^ (Table 1). Their mean BMD was −2.06 (±1.42) (T-score, lumbar). Thirty-three patients underwent three-level surgeries, and twelve patients underwent four-level surgeries (Table 2). The most common underlying disease in the study patients was hypertension. The mean operative time was 240 ± 42.2 min (74.6 ± 14.9 min per level) with a mean blood loss of 282.9 ± 167.1 cc. The duration of admission was 11.2 ± 4.9 days. The low back pain VAS score significantly decreased from 6.9 ± 1.1 to 2.1 ± 0.5 (*p* = 0.001) and leg pain VAS from 7.3 ± 1.0 to 1.8 ± 0.7 (*p* = 0.001). Leg pain showed immediate improvement after the surgery whereas back pain showed relatively gradual improvement during 6 months (Figure 6). The SF-12 score significantly improved from 32.2 ± 3.5 to 47 ± 4.2 (*p* = 0.001). There was one case of postoperative hematoma and one case of wound revision.

Compared to preoperative MRI images, cerebrospinal fluid (CSF) volume change and detachment of the rootlets show the immediate sufficient decompression of postoperative MRI images. Postoperative myelogram also showed improvement in signal blocking and recirculation of CSF.

**Figure 7:**
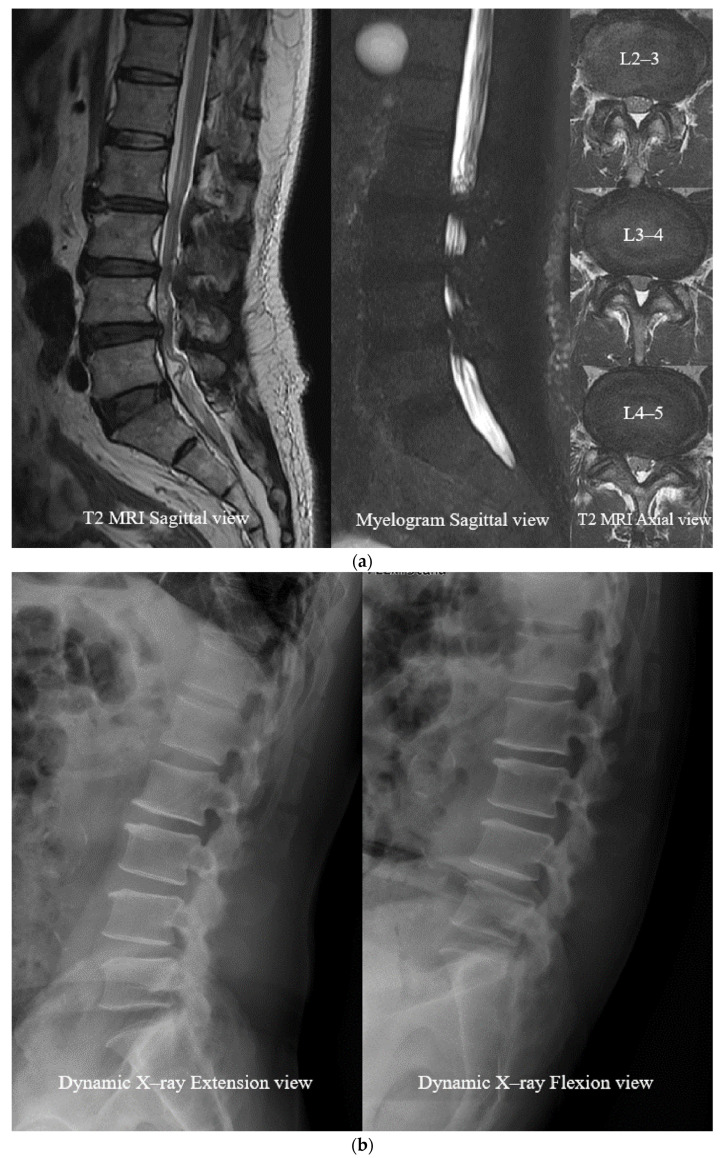

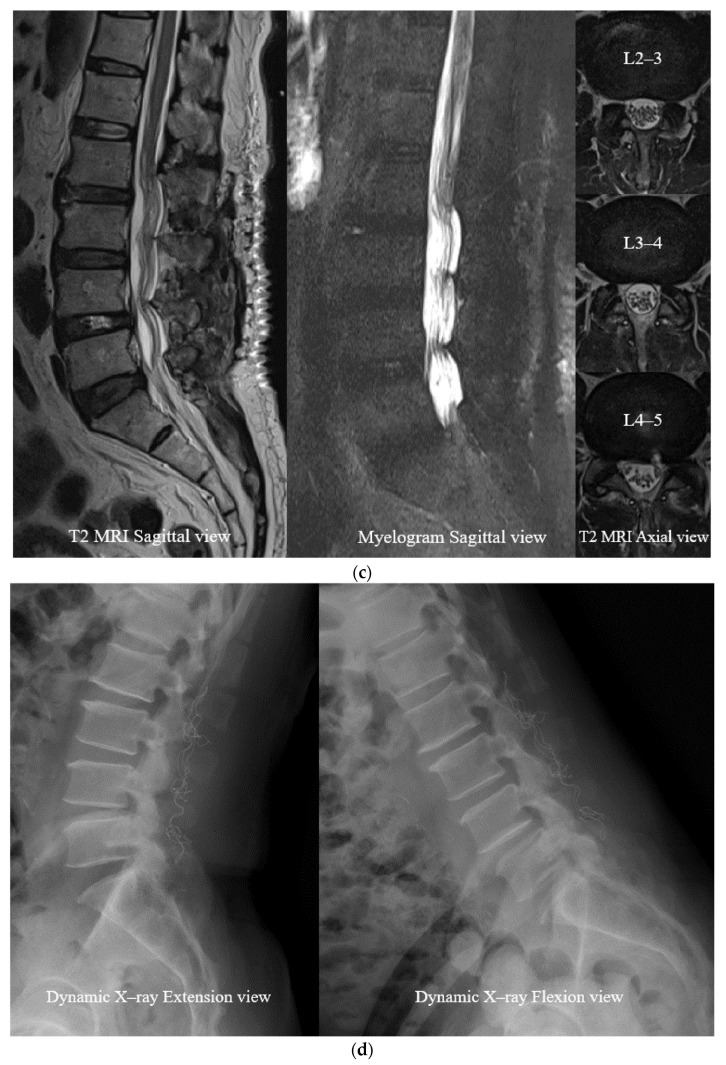

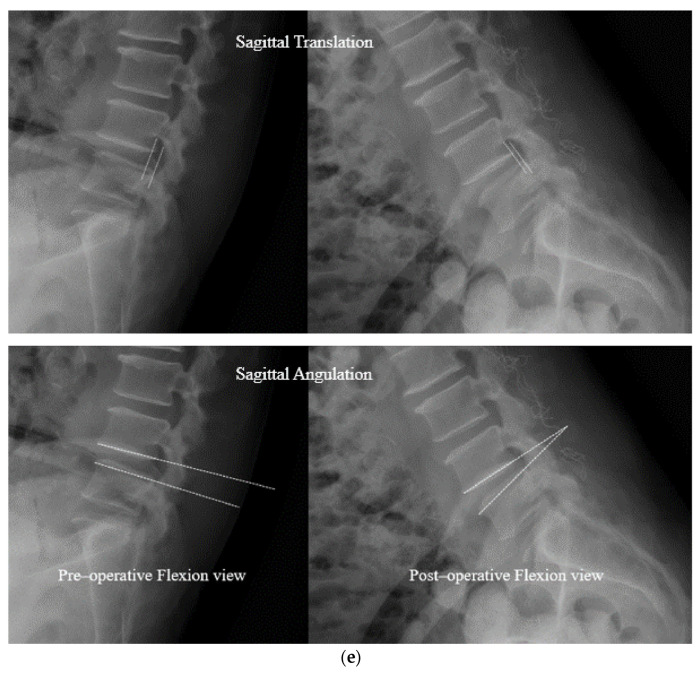
Case 2. A 79-year-old male patient suffered from low back pain and neurogenic claudication, which became aggravated after a 10 m walk. The physical examination revealed toe gait and hop test difficulties. (**a**) Preoperative MRI and myelogram: spinal stenosis at L2 to L5. (**b**) Preoperative dynamic X-ray: instability at L4–5. (**c**) Postoperative MRI: well-decompressed dural sac. (**d**) Postoperative dynamic X-ray: instability improvement at L4–5. (**e**) Sagittal translation decreased from 7.4 mm to 4.1 mm at L4–5. Sagittal angulation increased from −2.4° to 10.1°. Source: own composition. The small white dot line represents an extension of the posterior wall of the vertebra body and indicates the degree of listhesis. The large white dot line is an extension of the endplate line of the vertebra body and represents the degree of angulation.

Dynamic X-rays compared the pre-, postoperative (POD 1 week) flexion view X-rays. The results showed a sagittal translation decrease from 5.4 ± 2.1 mm to 3.1 ± 1.3 mm (*p* > 0.05) and a sagittal angulation increase from 1.0 ± 0.2° to 3.3 ± 1.2° (*p* > 0.05).

(Illustrated case 1; Figure 6, case 2; Figure 7).

## 4. Discussion

LSS is a very common degenerative disease [1]. If there is no improvement or the symptoms worsen even after sufficient conservative treatment, surgical treatment is considered. The most important process in surgical treatment is to decompress the nerve by removing the hypertrophied LF. However, looking at the pathophysiology of LSS, the hypertrophied LF is a form of degeneration cascade of the spine that often proceeds along with facet joint degeneration, disc degeneration, and anterior and posterior longitudinal ligament loosening, and it often occurs as a compensation reaction for instability [26]. Because of this pathogenic association, many LSSs are found together with degenerative spondylolisthesis and spinal instability. Therefore, when spine surgeons only remove the hypertrophic LF, the compressed nerve will be released and the symptoms of the lower extremities will be improved, but instability will tend to worsen. However, many spine surgeons currently focus only on removal of the hypertrophied LF and often overlook the instability of the index lesion. Therefore, many patients with LSS complain of lower back pain after surgery because the instability is not resolved even after decompression of the nerve. In addition, if the facet joint is removed too much in the process of decompression, it may actually worsen the instability iatrogenically [27].

Secondary delayed fusion surgery is a very common course in patients who continue to suffer from a remnant neurologic deficit after the first decompression surgery [28]. However, even this gold standard surgery has some fundamental limitations. First, when clinical symptoms such as back pain or leg pain remain after fusion surgery, there is no alternative surgical treatment other than conservative treatment. Second, compared to other simple spinal surgeries, fusion surgery carries some risks, including intraoperative bleeding, long operative time, and long recovery period. Thus, it is burdensome for patients and surgeons to perform on elderly patients with multilevel LSS [29,30]. Third, when secondary complications begin to appear, such as adjacent segment disease (ASD), pseudoarthrosis, and osteoporotic condition, more extensive deformity surgery is per-formed to correct the patient’s malalignment. Eventually, the patient’s QOL goes through a course that deteriorates worse than before surgery. Postoperative complications also in-crease substantially after long-level fusion surgery in elderly patients [31,32].

Because the human lifespan is limited, in order to delay this course of deterioration as much as possible, the process of supporting the original human spine structure should be improved so that the spine can be used as long as possible. For this, an intermediate stage of surgery is needed to fill the gap between decompression and fusion surgery. A treatment based on this concept involves “soft stabilization”.

First mentioned by Sengupta [33], the concept of soft stabilization has progressed widely. The ultimate goal of soft stabilization is not the concept of fusion that fundamentally blocks the joint movement of the index segment that causes pain but the concept of maintaining the existing joint movement as much as possible. There have been many attempts to treat spine lesions through alternative soft stabilization to overcome complications or long-term QOL degradation caused by fusion surgery. Various types of devices have been developed to date. However, most of these instruments have the common disadvantage of inflicting a violation on the bone through pedicle screw fixation [34,35,36,37,38,39,40].

OMD with ligament reconstruction introduced in this paper comprises new techniques for soft stabilization. This is achieved by simultaneously performing complete decompression and soft stabilization of the complete neural structure. Through the preceding process called OMD, the destruction of the facet structure, which is inevitably caused by spinous process splitting laminectomy, unilateral approach bilateral laminectomy, or bilateral laminectomy, can be minimized, and only the yellow LF can be exposed entirely. Through the interlaminar space, only the junction of the LF and the lamina is minimally drilled along the neural canal shape. This work can be performed in a relatively short time as it takes an average of 20 min per level as a result of this study (Table 2) even when per-forming it at multiple levels, and because it is not a fusion operation, it causes less bleeding during surgery. Although this paper is not a comparative study with the results of other fusion surgery, these operative results can be compared indirectly with those of other studies which demonstrated the operative results of posterior fusion surgery. Lei et al. [41] compared the blood loss and operation time of open posterior lumbar interbody fusion (PLIF) (168 ± 51 min, 445 ± 251 cc) and transforaminal lumbar interbody fusion (TLIF) (150 ± 50 min, 364 ± 181 cc). Yang et al. [42] compared the blood loss and operation time of MIS TLIF (179.0 ± 20.7 min, 355.3 ± 75.0 cc) and open TLIF (141.8 ± 18.8 min, 538.6 ± 129.5 cc). Compared with the results of other fusion papers, it can be estimated that this technique can shorten the amount of bleeding and operation time. Since it does not directly damage the bone or disc through a screw or cage, bleeding during surgery is reduced and the operative time is short. This can be a great advantage in that it can be implemented without much burden, even for elderly patients with LSS.

Compared with OMD with conventional midline-preserving bilateral laminectomy, the advantage is facet preservation. Bilateral laminectomy has no choice but to remove a significant portion of the facet due to the disturbance of the operation field caused by the spinous process. Iatrogenic injury to the facet joint may increase the risk of chronic back pain [43]. OMD has the great advantage of minimizing the destruction of the facet be-cause it alternately drills from the opposite side through the interlaminar space which is created by removing the interspinous ligament. To clarify the benefit of OMD, comparing the studies of other important clinical outcome parameters such as the Oswestry disability index (ODI), neurogenic claudication outcome score (NCOS), numeric rating scale (NRS), and Eu-roQol-5D (EQ-5D) between bilateral laminectomy and OMD will be needed in the future.

After the decompression process, the second step is to tie the intervertebral prosthetic ligament with strong tension to replace the weakened posterior ligament removed during the surgical procedure. This step plays a role in stabilizing the patient’s instability without fusion, which is also a very important part of the treatment for LSS. Our radiologic results showed improvement in instability after the surgery but were statistically insignificant. More studies are needed on the effect of using artificial ligaments to correct the instability that occurs during flexion. Nevertheless, as clearly observed on flexion X-rays before and after surgery in the preliminary case (Figure 6e and Figure 7e), there is some potential effect of ligament reconstruction to correct the instability. Approximately 6 weeks after surgery, soft tissue grows into the space between the filaments and is fixed more firmly than in the initial state.

Most of the patients did not complain of worsening of low back pain even until 6 months after the surgery (at final follow-up period). There are various causes of back pain, and a large portion is related to an injury of the interspinous ligament [44,45] and also to a collision of the spinous processes. This is commonly known as Baastrup’s disease [46,47]. OMD includes the process of removing a degenerated interspinous ligament, which can be the source of back pain. Also, the horizontal component of the artificial ligament is located between the two spinous processes and prevents the direct collision of them (Figure 5). For these reasons, artificial ligaments seem to show significant improvement not only for legs but also for low back pain.

Currently, in treatment for LSS, which cannot be resolved with conservative treatment, surgical strategies are considered including decompression and fusion surgery. Hyun et al. [48] mentioned that the trend of fusion surgery increased from 21.5% to 31.2% during 2004 to 2009. However, as previously described, fusion is the last surgical bulwark that a surgeon can perform, and it is not a perfect solution in itself, but surgery that can cause a fatal complication called ASD [49,50]. In addition, considering that many elderly patients have osteoporotic bone conditions, as shown in the results of our patients, the risk of instrument failure or pseudoarthrosis after fusion surgery is higher [51]. OMD with ligament reconstruction surgery is less invasive than multiple fusion surgery and can be performed successfully for the purpose of sufficient decompression with soft stabilization even for patients with old age and severe osteoporosis.

This study has several limitations. The retrospective design limits the level of evidence. The sample size was low (n = 42) and the follow-up period was short (6 months. There was a lack of a control group to compare ligament reconstruction with other surgical techniques. However, in our defense, the objective of this study was to provide a technical note and clinical outcomes following ligament reconstruction for multilevel stenosis. Future cohort studies with longer follow-up are required to further establish these findings.

In our experience, OMD with ligament reconstruction surgery serves to provide a fine balance between “decompression alone” and “fusion” surgery for multilevel stenosis. It adequately decompresses the nerves without significant removal of the lamina or facet joint. The artificial ligaments provide stability to the spine without actually fusing the motion segments. Thus, OMD with ligament reconstruction can negate the disadvantages of decompression alone (iatrogenic instability, worsening of pre-existing instability, back pain) and fusion (higher surgical invasiveness, complications such as pseudarthrosis, ASD, implant failure) while providing optimal outcomes. Unilateral laminotomy for bilateral decompression (ULBD) is a surgical alternative reported to have good results. However, it has been associated with iatrogenic instability, the failure of complete decompression, and non-improvement in back pain. The advantage of the less invasiveness of ULBD compared to OMD and ligament reconstruction can also be questionable for multilevel surgery. In terms of back pain, while some patients with neurogenic back pain show improvement after decompression alone, a major subset of patients fail to report any improvement and may require future surgery. We believe that OMD and ligament reconstruction surgery is a safe and effective surgical technique that offers more predictable outcomes compared to decompression alone and less invasiveness and complication rates compared to fusion surgery, especially in the context of multilevel stenosis. However, we do acknowledge that the findings of the current study do not provide comparisons with the outcomes of other surgical alternatives and future research is required in this regard.

## 5. Conclusions

Open midline decompression (OMD) with ligament reconstruction has been demonstrated to be a safe and effective surgical option for elderly patients suffering from multilevel lumbar spinal stenosis (LSS). This approach offers the significant advantage of achieving adequate nerve decompression while preserving the integrity of the facet joints with minimal destruction. Additionally, the soft stabilization provided by the artificial ligament serves as an innovative alternative to traditional fusion surgery, potentially reducing the risks associated with more invasive procedures.

For elderly patients, who often present with comorbidities and fragile bone conditions such as osteoporosis, this surgical technique represents a promising strategy. By maintaining spinal stability and functional mobility, OMD with ligament reconstruction may serve as a partial substitute for fusion surgery, addressing the challenges of multilevel LSS while minimizing postoperative complications and enhancing quality of life.

## Figures and Tables

**Table 1:** Patients’ demographic and clinical data (n = 45).

Variable		
Sex, M/F		18/27
Age, y		76.3 ± 3.79
Index level	3	33
	4	12
BMD spine, T-score *		−2.1 ± 1.4
Underlying disease	HTN ᵠ	33
	DM ¢	14
	Hyperlipidemia	17
	Thyroid disease	2
	Heart disease	6
	Liver disease	4
	Brain disease	6
	Lung disease	1
	Kidney disease	4
	Pancreatic disease	1
	Cancer	1
Smoker		11

* BMD: bone mineral density, as compared with individuals with maximum bone density. ᵠ HTN: hypertension. ¢ DM: diabetes mellitus. Thyroid disease: Hypothyroidism (n = 1), Nodule (n = 1). Heart disease: Angina (n = 3), aortic stenosis (n = 1), aortic insufficiency (n = 1), arrhythmia (n = 1). Liver disease: Hepatitis (n = 2), fatty liver (n = 2). Brain disease: Aneurysm (n = 2), Alzheimer’s disease (n = 2), vascular dementia (n = 1), infarction (n = 1). Lung disease: Asthma (n = 1). Kidney disease: Cyst (n = 2); chronic kidney disease (n = 2). Pancreatic disease: Cyst (n = 1). Cancer: Colon + uterine cancer (n = 1). Source: own composition.

**Table 2:** Patients’ operative data (n = 45).

Variable		n = 45
Level of surgery	Three levels	33
	Four levels	12
Total operative time (min) ᵠ		240 ± 42.2
Operative time per level ᵠ		74.6 ± 14.9
Perioperative fluid, cc		282.9 ± 167.1
Duration of admission, days		11.2 ± 4.9
Low back pain	Preoperative VAS score *	6.9 ± 1.1
	Postoperative VAS score	2.1 ± 0.5
	*p*-value	0.001
Leg pain	Preoperative VAS score *	7.3 ± 1.0
	Postoperative VAS score	1.8 ± 0.7
	*p*-value	0.001
SF-12 score ¢	Preoperative SF-12 score	32.2 ± 3.5
	Postoperative SF-12 score	47 ± 4.2
	*p*-value	0.001
Complication	Hematoma	1
	Wound suture	1

Pearson’s chi-square test, *p*-value < 0.05; statistically significant. Student’s *t*-test, *p*-value < 0.05; statistically significant. ᵠ Operative time: time from skin incision to waking up from anesthesia. * VAS: visual analog scale. ¢ SF-12: short-form 12-item questionnaire. Source: own composition.

## Data Availability

All data used during this study are available upon request from the corresponding author.
